# Guidelines for the use and management of long-acting injectable antipsychotics in serious mental illness

**DOI:** 10.1186/1471-244X-13-340

**Published:** 2013-12-20

**Authors:** Pierre Michel Llorca, Mocrane Abbar, Philippe Courtet, Sebastien Guillaume, Sylvie Lancrenon, Ludovic Samalin

**Affiliations:** 1 CHU Clermont-Ferrand, EA 7280, Clermont-Ferrand University, Clermont-Ferrand, France; 2 Department of Adult Psychiatry, CHU Caremeau, Nîmes, France; 3 CHRU Montpellier, INSERM U1061, Montpellier University, Montpellier, France; 4 Sylia-Stat, Paris, Bourg-la-Reine, France

**Keywords:** Guidelines, Long-acting injectable, Depot formulation, Antipsychotic, Schizophrenia, Bipolar disorder, Treatment

## Abstract

**Background:**

Long-acting injectable (LAI) formulations are not widely used in routine practice even though they offer advantages in terms of relapse prevention. As part of a process to improve the quality of care, the French Association for Biological Psychiatry and Neuropsychopharmacology (AFPBN) elaborated guidelines for the use and management of antipsychotic depots in clinical practice.

**Methods:**

Based on a literature review, a written survey was prepared that asked about 539 options in 32 specific clinical situations concerning 3 fields: target-population, prescription and use, and specific populations. We contacted 53 national experts, 42 of whom (79%) completed the survey. The options were scored using a 9-point scale derived from the Rand Corporation and the University of California in the USA. According to the answers, a categorical rank (first-line/preferred choice, second-line/alternate choice, third-line/usually inappropriate) was assigned to each option. The first-line option was defined as a strategy rated as 7–9 (extremely appropriate) by at least 50% of the experts. The following results summarize the key recommendations from the guidelines after data analysis and interpretation of the results of the survey by the scientific committee.

**Results:**

LAI antipsychotics are indicated in patients with schizophrenia, schizoaffective disorder, delusional disorder and bipolar disorder. LAI second-generation antipsychotics are recommended as maintenance treatment after the first episode of schizophrenia. LAI first-generation antipsychotics are not recommended in the early course of schizophrenia and are not usually appropriate in bipolar disorder. LAI antipsychotics have long been viewed as a treatment that should only be used for a small subgroup of patients with non-compliance, frequent relapses or who pose a risk to others. The panel considers that LAI antipsychotics should be considered and systematically proposed to any patients for whom maintenance antipsychotic treatment is indicated. Recommendations for medication management when switching oral antipsychotics to LAI antipsychotics are proposed. Recommendations are also given for the use of LAI in specific populations.

**Conclusion:**

In an evidence-based clinical approach, psychiatrists, through shared decision-making, should be systematically offering to most patients that require long-term antipsychotic treatment an LAI antipsychotic as a first-line treatment.

## Background

Schizophrenia and bipolar disorder are examples of some chronic illnesses for which there exists a high risk of relapse associated with major functional consequences. The pharmacologic strategy can be considered as the cornerstone of the treatment for these patients. Compliance is often mediocre with deleterious consequences [1]. For example, the majority of patients with schizophrenia (84%) discontinue their index antipsychotic during the follow-up period [2] and in the long-term perspective, 40 to 50% seem to be non-compliant [3], with no real difference in terms of adherence between first-generation antipsychotics (FGA) and second-generation antipsychotics (SGA) [4].

Long-acting injectable (LAI) antipsychotics have been part of the pharmacopoeia for over 40 years. Various meta-analyses highlight their interest as a relapse prevention strategy in schizophrenia [5-7]. With regards to non-adherence, most of the guidelines and algorithms (except PORT 2009) state that depot antipsychotics are an effective approach [8-10], with some guidelines actually recommending that switching the antipsychotic formulation from oral to depot should be considered in maintenance treatment [11].

Nevertheless, depot formulations are still poorly used overall in routine practice, with prescription rates in different countries generally no more than 25% [12,13]. However, use of the depot forms varies between countries. Prescription rates are higher in France (23.5%) [14] and the United Kingdom (29%) [12] compared to other European countries. Several factors that deter psychiatrists from using depot forms have been identified, stemming from mistaken beliefs about good adherence, patient refusal, perceived coercion or a presumed risk of lower tolerance [13,15]. At a practical level, psychiatrists need to be confident and competent in presenting patients with sufficient information to enable them to make an informed choice about whether to accept oral or LAI medication or neither. We state that the development and diffusion of specific guidelines, addressing all the aspects of the use of LAI antipsychotics, will increase clinicians’ perceived competence. It will also help to increase the percentage of patients to whom LAI antipsychotics will be offered by psychiatrists as a therapeutic option.

The objective of these guidelines is to propose a prescription framework to clinicians for the use of a specific formulation of antipsychotics (LAI) in diverse therapeutic indications and specific clinical situations. The aim is to allow clinicians to offer the most appropriate pharmaceutical strategies to the patients and to facilitate the use of LAI antipsychotics in clinical practice. The recommendations presented here from a consensus-based guidelines methodology (Formal Consensus Guidelines) are based on scientific data and the consensus of a panel of experts.

## Methods

### Questionnaire development

Initially, we performed an analysis and a literature review concerning the indications and the use of LAI antipsychotics. A literature search using the keywords “antipsychotic”, “neuroleptic”, “first-generation antipsychotic”, “atypical antipsychotic”, “second-generation antipsychotic”, “long-acting injectable”, “depot”, “depot neuroleptic” was performed in PubMed and EMBASE to find all the relevant studies published. Additional references were identified from http://www.fda.gov and http://www.ema.europa.eu.

Data from all of these sources was discussed and an overview of the current evidence has been graded and summarized using the French National Authority for Health (HAS) “levels of evidence” criteria [16].

Following this first step, the scientific committee (PML, LS, MA, PC, SG, SL) created a questionnaire consisting of 32 questions that covered 539 therapeutic options. The 32 questions were regrouped into 3 areas that were judged as essential:

Target-population: Description of the different indications of the LAI antipsychotics and of the most appropriate period of the illness to introduce the treatment.

Prescription and use: Choice of the molecule, methods of introduction, specific strategies depending on the psychiatric disorder or co-morbidities, and treatment monitoring.

Specific population: Use of LAI antipsychotics in pregnant women, elderly patients, subjects in a precarious situation, and subjects having to be treated in a prison establishment.

This questionnaire was designed to be completed by an experts’ panel. The time required for its administration was estimated at around 3 hours.

At the time of development, all the LAI antipsychotics available in France were proposed as therapeutic options (Table 1). They were regrouped into 2 categories:

Long-acting injectable first-generation antipsychotics (LAI FGA).

Long-acting injectable second-generation antipsychotics (LAI SGA).

**Table 1:** LAI antipsychotics available in France (when the survey was completed)

**LAI second-generation antipsychotics**	**Risperidone microsphere**
	**Olanzapine pamoate**
**LAI first-generation antipsychotics**	**Haloperidol decanoate**
	**Zuclopenthixol decanoate**
	**Flupentixol decanoate**
	**Fluphenazine decanoate**
	**Pipotiazine palmitate**

Note: as paliperidone palmitate had a marketing authorization date after the development of these guidelines, it could not be taken into account.

This artificial separation FGA/SGA is not consensual due to their heterogeneous profiles of efficacy and tolerability (especially for SGA) [17,18]. However, we maintained both these categories to facilitate the elaboration, the reading and the understanding of this guideline.

### Rating scale

The experts were able to express their level of agreement or disagreement for each question. The rules that describe, on the one hand the agreement (or the disagreement), and on the other hand the degree of convergence of the expert opinions, were predefined.

**Figure 1:**
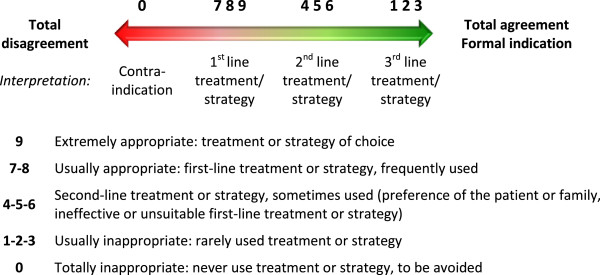
Rating scale.

Each expert answered each question with the help of a graduated scale from 0 to 9 (0 meaning a “total disagreement” or “a formal contraindication” and 9 indicating a “total agreement” or “a formal indication”) (Figure 1).

This scale is derived from a variation of the “Nominal Group” method, developed by the *Rand Corporation* and the University of California in the USA (“*RAND*/*UCLA appropriateness rating method*”).

The scale has the advantage of being well standardized, used in medicine and of having been published [19].

### Expert selection

The Scientific Committee (Appendix 1) selected 53 French psychiatrists considered to be experts in the use of LAI antipsychotics.

The selection criteria were:

Clinical activity in the field of schizophrenia or bipolar disorder.

Publication (s) or communication (s) of research work in the field of LAI antipsychotics in national or international congresses.

Each expert was consulted individually for his or her expertise using the supplied questionnaire. A follow-up was established to ensure, essentially, the sending and returning of these documents. A reminder of the expert's mission was made. Written commitment from each expert was requested. The experts participating in this project were not paid.

### Data analysis

The answers to each question or proposition were quantitatively analyzed (number of answers, median, mean, standard deviation, minimum, maximum) (Table 2).

First-line treatment/strategy was defined if at least 50% of the answers to the question were in the zone 7–9 and less than 20% were in the zone 0. The treatment/strategy of choice was kept if at least 50% of the experts had rated it 9.

Second-line treatment/strategy was defined if less than 50% of the answers to the question were in the zone 7–9, at least 50% were in the cumulated zones 7–9 and 4–6, and less than 20% were in the zone 0.

Third-line treatment/strategy was defined if less than 50% of the answers to the question were in the cumulated zones 7–9 and 4–6, and less than 20% were in the zone 0.

Contraindication was defined if at least 50% of the answers were in the zone 0.

**Table 2:** Data analysis

**Percentage of answers in the zones**				
**0**	**1-3**	**4-6**	**7-9**	
< 20%	-	< 50%	≥ 50%	→ First-line treatment/strategy
< 20%	< 50%	≥ 50% and < 100%		→ Second-line treatment/strategy
< 20%	-	< 50%		→ Third-line treatment/strategy
≥ 50%	-	-	-	→ Contraindication

For all other cases (-), the question was considered as non-consensual.

The following rules were used to conclude the analyzed therapeutic strategy.

**Figure 2:**
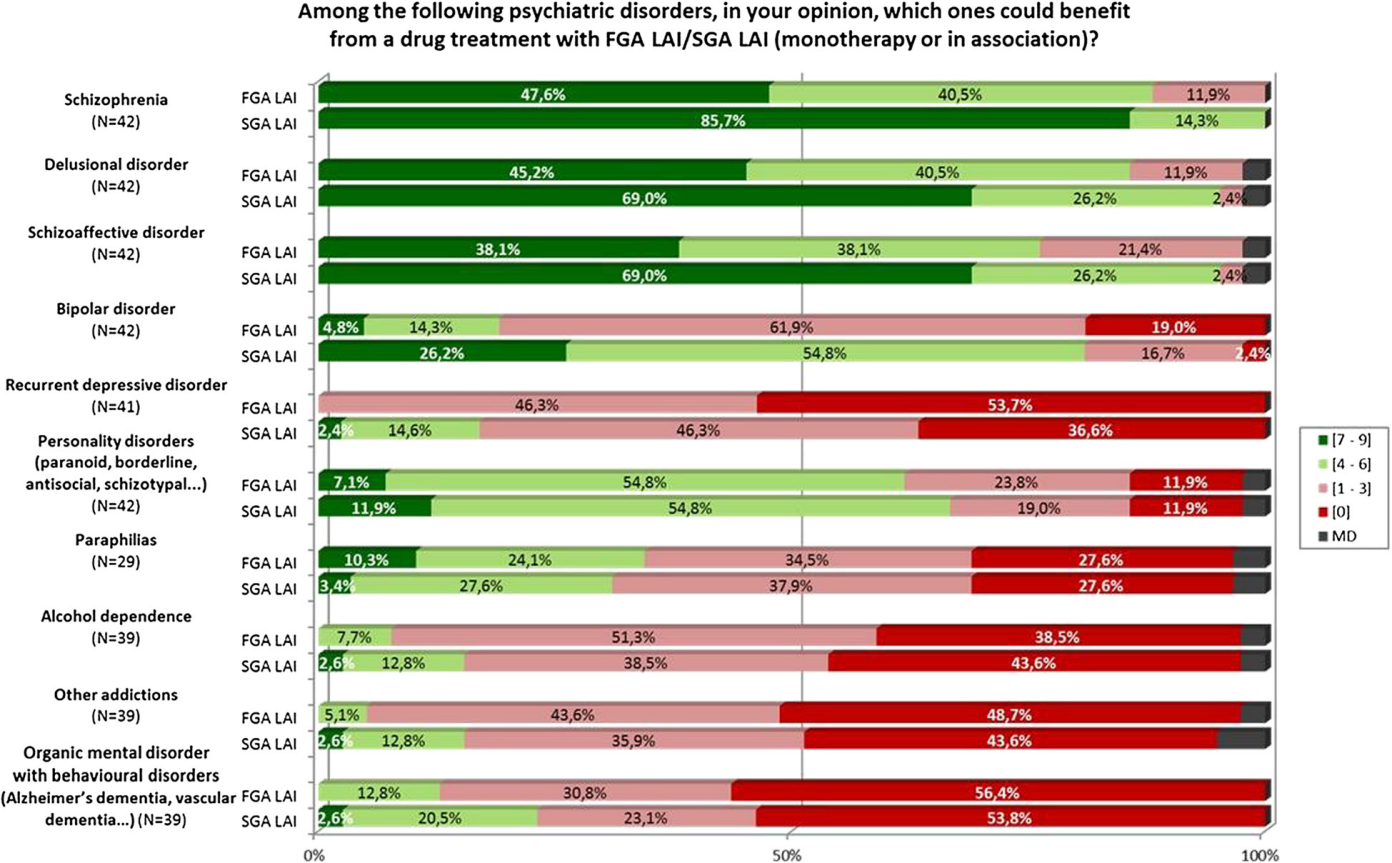
Graphic results of the question about indications for use of LAI.

For all other cases the question was considered as non-consensual. An example is given in Figure 2. The results were interpreted by the scientific committee and permitted the development of the recommendations. An independent committee (Appendix 1) validated the final version of recommendations (EH, CL, PT). Two members of the scientific committee elaborated the final document (LS, PML).

## Results

The following sections summarize the key recommendations from the guidelines after data analysis and interpretation of the results of the survey by the scientific committee. The complete database (with questions and answers) is available on the website http://www.afpbn.org. However, several examples of questions, with the experts’ answers, are presented here to facilitate understanding of the results section.

### Description of the expert population

Forty-two experts completed the questionnaire (Appendix 2), representing 79% of those contacted. The reasons for the non-participation of the remaining 11 experts were that they had either too much consultancy work or insufficient availability to reply within the time limits. The socio-demographic data and professional activities of the experts’ panel are presented in Table 3.

**Table 3:** Socio-demographic data and professional activities of the experts’ panel (N = 42 experts)

**Age (years)**	N	42
	Mean ± SD	46.81 ± 9.82
	Min; Max	31; 63
	Median	46
**Years of practice**	N	41
	Mean ± SD	17.29 ± 10.20
	Min; Max	2; 37
	Median	16
**Treatment of patients in outpatients**	N	41
	Mean ± SD	68.90 ± 22.43
	Min; Max	25; 100
	Median	75
**Treatment of patients in hospital**	N	41
	Mean ± SD	31.10 ± 22.43
	Min; Max	0; 75
	Median	25
**During the last 5 years, in the field of LAI FGA/LAI SGA**	N	42
	Clinical activity	42 (100.0%)
	Research projects	18 (42.9%)
	Publications	12 (28.6%)
**Communications**	N	36
	Conferences	22 (61.1%)
	Congress	24 (66.7%)
	Teaching	22 (61.1%)

### Target population

#### Indications

Indications for the use of LAI FGA and LAI SGA are summarized in Table 4.

**Table 4:** LAI FGA and LAI SGA indications according to the DSM-IV-TR criteria

**LAI FGA**	**LAI SGA**
**1**^**st **^**line treatment**	
	Schizophrenia
	Delusional disorder
	Schizoaffective disorder
**2**^**nd **^**line treatment**	
Schizophrenia	Bipolar disorder
Delusional disorder	Personality disorder
Schizoaffective disorder	
Personality disorder	

The relevant question from the survey with the experts’ answers are given in Figure 2.

LAI SGA are recommended (in monotherapy or combination):

as 1^st^ line treatment in schizophrenia, delusional disorder and schizoaffective disorder.

as 2^nd^ line treatment in bipolar disorder and personality disorders.

They are contraindicated in organic mental disorders with behavioural disorders (Alzheimer’s disease, vascular dementia).

LAI FGA are recommended (in monotherapy or combination):

as 2^nd^ line treatment in schizophrenia, delusional disorder, schizoaffective disorder and personality disorders.

They are contraindicated in recurrent depressive disorder and in organic mental disorders with behavioural disorders.

#### Most appropriate introduction period during the illness

The most appropriate period for the introduction of LAI FGA and SGA are summarized Table 5.

**Table 5:** Use of LAI FGA and LAI SGA according to the period of the illness

**LAI FGA**	**LAI SGA**
**Schizophrenia**	
LAI FGA are not recommended in the initial phase of the disorder.	Very early introduction of LAI SGA is recommended (eventually from the 1^st^ psychotic episode).
LAI FGA can be used during the maintenance treatment in the case of the efficacy of the oral form and when the benefit/risk ratio is considered as satisfactory.	It is recommended that an LAI SGA be introduced from the 1^st^ recurrent psychotic episode (if the patient was not treated with an LAI antipsychotic).
**Bipolar disorder**	
LAI FGA are not recommended.	LAI SGA are not recommended in the initial phase of bipolar disorder.

Only LAI SGA are considered as a therapeutic option during the initial phase of schizophrenic illness:

They are recommended from the first psychotic episode.

Their introduction from the first recurrent psychotic episode is also recommended (if the patient was not treated with an LAI antipsychotic).

LAI FGA are not recommended during the early course of schizophrenia (i.e. in a patient who has been newly diagnosed with schizophrenia and who has had no previous antipsychotic treatment). They must be used as maintenance treatment during the long-term evolution of the illness in the case of efficacy of the corresponding oral formulation and when the benefit/risk ratio is considered as satisfactory.

#### Choice criteria for an LAI FGA or LAI SGA according to the clinical characteristics of patient

The different clinical criteria for the use of LAI FGA and SGA are presented in Table 6.

**Table 6:** Indications of LAI FGA and LAI SGA according to clinical characteristics of the illness

**Schizophrenia**			**Bipolar disorder**		
**1st line**	**LAI FGA or LAI SGA**	Frequent relapses Non-adherence (partial/full) Hazard risk for others Low insight Patient preference Positive depot experienced	**1st line**		Non-adherence (partial/full) Patient preference Positive depot experienced
	**LAI SGA**	Cognitive deficits Social isolation		**LAI SGA**	
**2**^**nd**^** line**	**LAI FGA or SGA**	Positive symptoms	**2**^**nd**^** line**		BD I Manic polarity Rapid cycler Hazard risk for others Low insight
	**LAI SGA**	Negative symptoms Suicidal risk			

#### Schizophrenia

The preferential choice criteria for an LAI formulation (as 1^st^ line treatment) in patients with schizophrenia are:

Patients presenting frequent relapses, poor adherence or non-acceptance of a long-term treatment.

LAI FGA or LAI SGA are recommended as 1st line treatment. In the case of poor observance, LAI SGA are considered as the treatment of choice.

Patients presenting dangerous behavior.

LAI FGA and LAI SGA are recommended as 1^st^ line treatment.

Patients presenting a low level of insight about illness and need for treatment.

LAI FGA and LAI SGA are recommended as 1^st^ line treatment.

Patients wishing treatment by LAI antipsychotic and/or having a history of effective treatment by LAI FGA or LAI SGA.

LAI FGA or LAI SGA are recommended as 1^st^ line treatment.

Patients presenting cognitive impairment with an impact on their functioning.

LAI SGA are recommended as 1^st^ line treatment. LAI FGA are not recommended.

Socially and family isolated patients.

LAI SGA as 1^st^ line treatment (LAI FGA as a 2^nd^ line treatment) are recommended for patients with poor social and family support.

Patients receiving outpatient care without consent.

When a compulsory outpatient care program is planned, LAI SGA as 1^st^ line treatment (LAI FGA as 2^nd^ line treatment) are recommended.

The experts failed to reach a favorable consensus for the preferential use of an LAI formulation (as 1^st^ line treatment) for the following groups. They just specified the preferential category of LAI (FGA or SGA) for these groups.

Patients presenting a predominant clinical dimension.

The prevalence of positive or negative symptoms is not a specific factor in choosing to use a depot treatment.

If a depot treatment is chosen:

* LAI SGA and LAI FGA are recommended (as 2^nd^ line treatment) for clinical forms where positive symptoms prevail.

* Only LAI SGA are recommended (as 2^nd^ line treatment) in cases of predominant negative symptoms.

Patients presenting a high level of suicide intention.

Only LAI SGA are considered (as 2^nd^ line treatment) for patients presenting suicidal behavior during acute episodes.

Patients presenting a high level of insight about their illness.

A high level of insight about the illness can be an indication for proposing an LAI SGA as a 2^nd^ line treatment. LAI FGA are not recommended in cases of high levels of insight about the illness.

#### Bipolar disorder

The preferential choice criteria for an LAI formulation (as 1^st^ line treatment) in bipolar patients are:

Patients presenting poor adherence with non-acceptance of a long-term oral treatment.

LAI SGA are recommended as a 1^st^ line treatment (in monotherapy or in combination).

Patients wishing for an LAI SGA treatment and/or having a history of effective treatment with LAI SGA for bipolar disorder symptoms.

Irrespective of the clinical situation, LAI FGA are never recommended as maintenance treatment for bipolar disorder.

The experts failed to reach a favorable consensus for the preferential use of a LAI formulation (as 1^st^ line treatment) for the following groups. They just specified the preferential category of LAI (FGA or SGA) for these groups.

Patient presenting particular clinical characteristics.

Owing to the medications currently available, LAI SGA are recommended (as 2^nd^ line treatment) in patients presenting a type I bipolar disorder and/or a predominant manic polarity and/or rapid cycles.

Patients presenting a dangerous behavior or a history of impulsive behavior.

LAI SGA are recommended as 2^nd^ line treatment.

Patients presenting a low level of insight about the need for treatment.

LAI SGA are recommended as 2^nd^ line treatment.

#### Benefit/risk balance for LAI FGA and LAI SGA depending on the psychiatric disorder

##### In patients with schizophrenia

**Figure 3:**
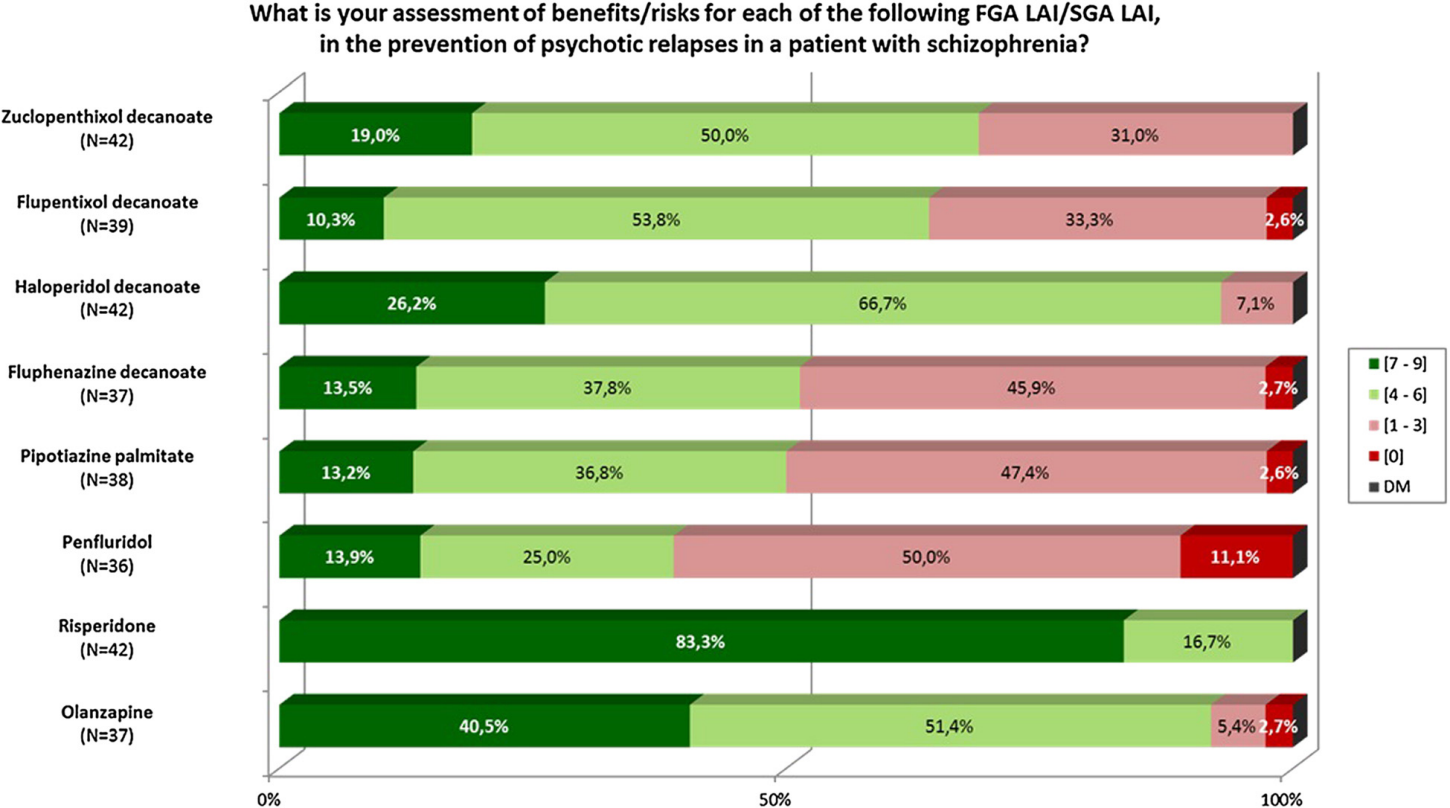
Graphic results of the question about benefit/risk balance for LAI FGA and LAI SGA in schizophrenic patients.

The assessment of the benefit/risk ratio for each LAI formulation in the preventive treatment of psychotic recurrence is presented in Table 7. The relevant question from the survey with the experts’ answers are given in Figure 3.

**Table 7:** Benefit/risk ratio for LAI FGA and LAI SGA in schizophrenia

	**Prevention of psychotic recurrence**
**1**^**st **^**line treatment**	Risperidone LAI
**2**^**nd **^**line treatment**	Olanzapine pamoate
	Haloperidol decanoate
	Zuclopenthixol decanoate
	Flupentixol decanoate
	Fluphenazine decanoate
	Pipotiazine palmitate

The molecule ranking appears to be directly linked to the tolerance level for each LAI antipsychotic. LAI SGA are recommended as 1^st^ line treatment except for olanzapine pamoate.

##### In patients with bipolar disorder

Only two LAI SGA are recommended as 2^nd^ line treatment: risperidone microsphere and olanzapine pamoate (Table 8). LAI FGA are contraindicated as maintenance treatment of bipolar disorder.

**Table 8:** Benefit/risk ratio for LAI FGA and LAI SGA in bipolar disorder

	**Prevention of manic recurrence**	**Prevention of depressive recurrence**
**1**^**st**^**-line treatment**	-	-
**2**^**nd**^**-line treatment**	*In monotherapy or in combination with a mood stabilizer*	*Always in combination with a mood stabilizer*
	Risperidone LAI	Risperidone LAI
	Olanzapine pamoate	Olanzapine pamoate

### Procedures for prescribing and use

#### Patients stabilized by an antipsychotic treatment

##### Switch from an oral form antipsychotic (FGA or SGA) to an LAI form

First-line strategy is to start with the antipsychotic oral form for the length of time required to obtain an effective dose and good tolerance before switching to the LAI form.

Note. Only risperidone microspheres have the pharmacokinetic characteristics that imperatively require an initial oral supplement.

The prescription of LAI SGA must be made while taking into account the pharmacokinetic characteristics of each product.

The dose of the introduced LAI form will correspond to the equivalent of the used oral dose (strategy of choice).

##### Switch from an LAI antipsychotic (FGA or SGA) to another LAI antipsychotic

First-line strategy is to introduce the new LAI antipsychotic after the discontinuation of the current LAI FGA or LAI SGA (when the time since the last injection corresponds to the interval between 2 injections).

In 2^nd^ line strategy, the switch from the current LAI FGA or LAI SGA to the new LAI SGA is recommended directly after having given an oral test dose of the newly introduced SGA LAI in order to eliminate any hypersensitivity.

The initial dose for the oral form or for the new LAI SGA will correspond (if possible) to an equivalent dose of the previous LAI FGA or LAI SGA (1^st^ line strategy).

#### Practical procedures for the introduction and for the injection reminders

In order to help with the acceptance and understanding of the benefits of an LAI treatment, it is unanimously recommended by the experts (strategy of choice) to convey to the patient specific information concerning both the advantages and inconveniences of the FGA and SGA LAI, which are being considered, in the framework of shared decision-making.

During the introduction of the treatment, initiation of the LAI form is recommended before the end of a full-time hospitalization for an acute episode (strategy of choice). Introduction of LAI antipsychotics can also be considered during outpatient care (as 2^nd^ line strategy).

The 1^st^ line strategy of performing the injections during the maintenance treatment in outpatients is to coordinate the follow-up psychiatric consultations with the dates of the injections. The injections can also be performed by a nurse in a hospital day care unit or at home (as 1^st^ line strategy).

Note: these injection procedures are not applicable to olanzapine pamoate as this treatment requires specific post-injection monitoring in a hospital.

In order to improve patient compliance, it is recommended that the following reminder techniques are put in place:

**1**^**st **^**line strategies, using telephone reminders and agenda given to the patient (follow-up diary).**

**2**^**nd **^**line strategies, by letter or eventually by text messages.**

The prevention of local complications requires the injections to be performed:

deep intramuscularly (gluteal or deltoid muscle) (strategy of choice).

by changing the injection site each time (as 1^st^ line strategy).

by proposing a local transdermal anaesthetic (cream or patch) before the injection in order to reduce the pain at the injection site (as 2^nd^ line strategy).

#### Specific therapeutic strategies according to the psychiatric disorder or its co-morbidities

#### Schizophrenia and delusional chronic disorder

Acute psychotic episode with LAI FGA or LAI SGA treatment

**Figure 4:**
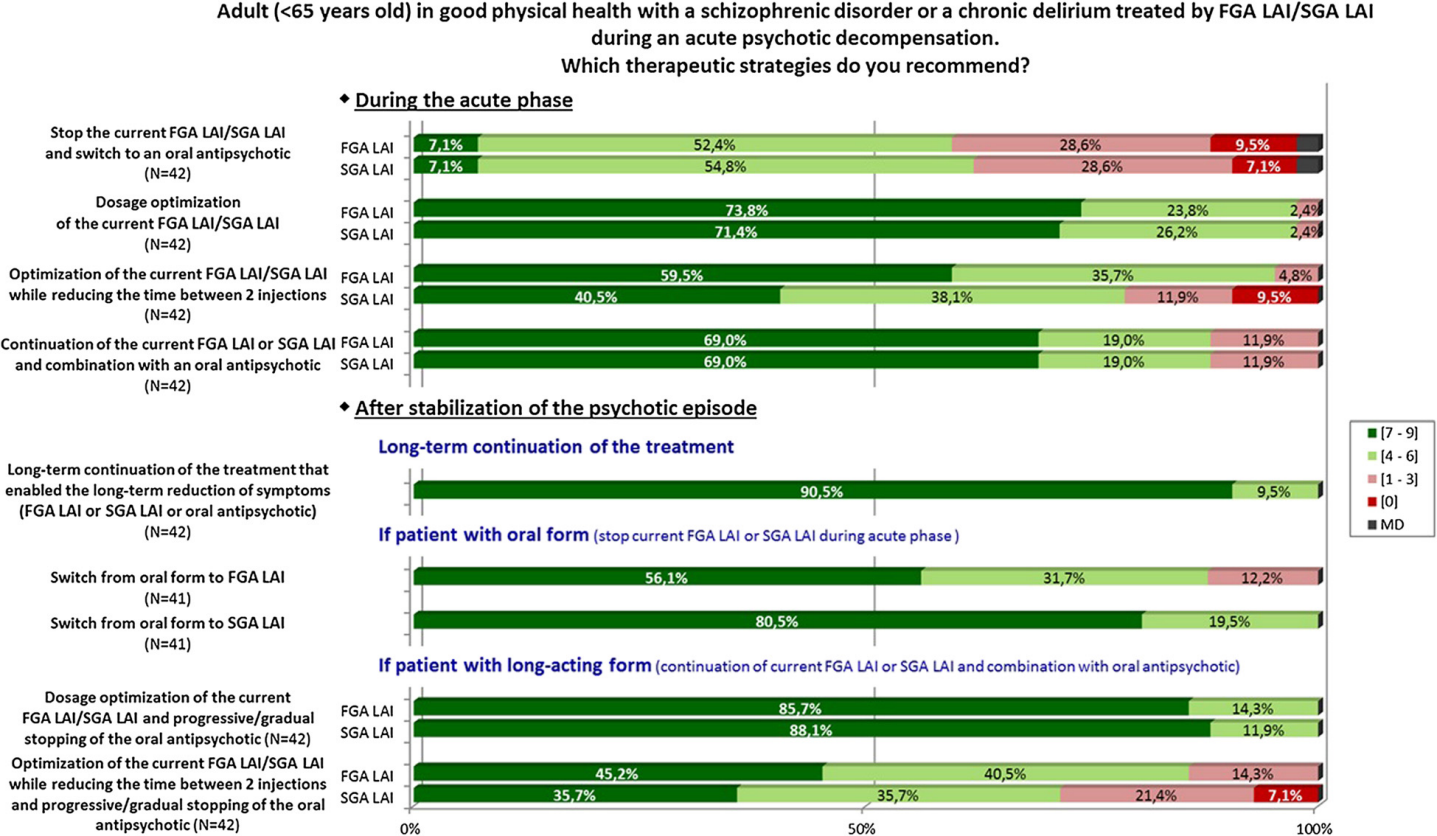
Graphic results of the question about therapeutic strategies during an acute psychotic episode.

The relevant question from the survey with the experts’ answers are given in Figure 4.

#### - In the acute phase

Several therapeutic adaptations are recommended as 1^st^ line strategies:

Optimization of the current LAI antipsychotic.

* either dose optimization of the current LAI FGA or LAI SGA by increasing the dose while monitoring tolerance.

* or for LAI FGA: reduction of the time between 2 injections.

Combination of an oral antipsychotic with the current LAI antipsychotic.

The discontinuation of the current LAI antipsychotic and the switch to an oral antipsychotic in the acute phase is only recommended as 2^nd^ line strategy.

#### - After stabilization of the psychotic episode

It is recommended to continue as maintenance treatment the therapeutic strategy that allowed the reduction of symptoms and the stabilization of the episode (strategy of choice).

In the case of a switch to an oral antipsychotic treatment during the acute phase, switching to an LAI formulation as maintenance treatment is recommended as the 1^st^ line strategy.

In the case of the combination of an oral antipsychotic and an LAI antipsychotic in the acute phase, optimizing the dose of the LAI antipsychotic and progressively discontinuing the oral antipsychotic while monitoring the clinical state is recommended as the 1^st^ line strategy.

#### Residual symptoms with LAI antipsychotics justifying a reassessment

It is successively recommended:

in 1^st^ line strategies: to optimize the treatment by LAI FGA or LAI SGA.

* by dose optimization of the current LAI antipsychotic by increasing the dose while monitoring tolerance.

* or for LAI FGA: by reducing the time between 2 injections.

in 2^nd^ line strategies.

* either through a combination of an oral antipsychotic with the current LAI antipsychotic.

* or by changing the current LAI FGA or LAI SGA for another LAI antipsychotic (preferably a LAI SGA).

#### Bipolar disorder

#### Manic episode with LAI SGA

-In the acute phase

If monotherapy is ongoing, it is successively recommended:

in 1^st^ line strategy: to combine the current LAI SGA with an oral anti-manic mood stabilizer (without recommendation of a specific medication).

in 2^nd^ line strategies.

* to optimize the dose of the current LAI SGA by increasing the dose while monitoring tolerance.

* or to discontinue the current LAI SGA and switch to an oral anti-manic mood stabilizer (without recommendation of a specific medication).

*If bitherapy is ongoing* (*LAI SGA* + *lithium or anticonvulsant*), it is successively recommended:

in 1^st^ line strategy: to optimize the dose of the oral anti-manic mood stabilizer.

in 2^nd^ line strategies.

* either to combine the current LAI SGA with another oral anti-manic mood stabilizer (without recommendation of a specific medication).

* or to optimize the dose of the current LAI SGA by increasing the dose while monitoring tolerance.

* or to discontinue the current LAI SGA and switch to a bitherapy of oral anti-manic mood stabilizers (without recommendation of a specific medication).

* or to continue the current treatment and combination with a 2^nd^ oral anti-manic mood stabilizer (without recommendation of a specific medication).

* or to continue the current treatment and electroconvulsive therapy (ECT) administration.

#### - After stabilization of the manic episode

It is recommended to continue as maintenance treatment the therapeutic strategy that allowed the reduction of the symptoms and the stabilization of the episode (no precision on the duration) (strategy of choice).

#### Depressive bipolar episode with LAI SGA

#### - In the acute phase

*If monotherapy is ongoing*, it is successively recommended:

in 1^st^ line strategy: to combine the current LAI SGA with an oral mood stabilizer with antidepressant effect (i.e. lamotrigine, quetiapine, lithium).

in 2^nd^ line strategies.

* either to optimize the dose of the current LAI SGA by increasing the dose while monitoring tolerance.

* or to combine the current LAI SGA with an oral antidepressant or with a series of ECT.

* or to discontinue the current LAI SGA and switch to an oral mood stabilizer with antidepressant effect.

***If bitherapy is ongoing *** **(*LAI SGA*** **+** ***antidepressant*), it is successively recommended:**

in 1^st^ line strategies.

* either to optimize the dose of the current oral antidepressant by increasing the dose while monitoring tolerance.

* or to continue the combination of a LAI SGA with an antidepressant and combination with an oral mood stabilizer with antidepressant effect.

in 2^nd^ line strategies.

* either to combine another oral antipsychotic with the current LAI SGA.

* or to optimize the dose of the current LAI SGA by increasing the dose while monitoring tolerance.

* or to discontinue the current LAI SGA and switch to a bitherapy of oral mood stabilizers and oral antidepressant.

* or to continue the current treatment and ECT administration.

#### - After stabilization of the depressive episode

In the 1^st^ line strategy, it is recommended to continue as maintenance treatment the therapeutic strategy that allowed the reduction of symptoms and the stabilization of the clinical state (no precision of the duration).

In the 2^nd^ line strategy, in the case of the combination of an oral antidepressant with an LAI SGA in the acute phase, it is recommended to optimize the dose of the LAI SGA and to progressively discontinue the oral antidepressant, depending on the clinical state.

#### Psychiatric co-morbidities associated with a schizophrenic or bipolar disorder with an LAI antipsychotic

Manifestations of anxiety (structured or non-structured)

It is recommended in 1^st^ line treatment to associate an oral benzodiazepine, and in 2^nd^-line treatment to combine an antidepressant (as first-line treatment, an SSRI or SNRI).

#### Addiction to a psychoactive substance (alcohol, opiates…)

Treatment by LAI SGA or LAI FGA can be continued. The prescription of opiate substitutes (buprenorphine or methadone) (1^st^ line strategies) or disulfiram, acamprosate or naltrexone (2^nd^ line strategies) depending on the addiction, is possible with LAI antipsychotics.

#### Procedures for follow-up and monitoring

#### Pre-therapeutic LAI antipsychotic summary

As 1^st^ line strategies, it is recommended to systematically search for the following *clinical elements*:

Personal and family medical history (diabetes, dyslipidaemia).

Healthy lifestyle (eating habits, physical activity, substance use, smoking).

Weight, Body Mass Index calculation, umbilical circumference.

Blood pressure.

It is recommended to perform the following *paraclinical checkups*:

•1^st^ line paraclinical exams:

Complete blood count, blood electrolyte (+ urea, creatinine, fasting glucose).

Liver function tests.

Lipid profile.

Beta hCG.

Electrocardiogram.

•Paraclinical exams depending on the clinical state of patient (as 2^nd^ line):

Thyroid function test.

Prolactinaemia.

Electroencephalogram.

All the experts recommended informing the patient and the family of the risks of adverse event occurrence (metabolic, neurological…) as well as providing hygiene and diet advice (balanced diet, regular physical activity, reduction or help in stopping substance use…) (strategy of choice).

#### Monitoring procedures

Clinical and paraclinical monitoring of LAI antipsychotics is the same as for oral antipsychotics

The specific monitoring frequency will depend on the risk factors found in the patient and on the clinical signs that appear during the treatment as well (1st-line strategies).

### Specific populations

#### Women during pregnancy

*In the case of planned pregnancy in a woman treated with LAI antipsychotic*

The experts failed to reach a favorable consensus for 1st-line strategies in this clinical situation. As a 2^nd^ line strategy, it is recommended to discontinue the current LAI antipsychotic and switch to the oral form (at the minimum effective dose).

### In the case of discovering a pregnancy

In the 1^st^/2^nd^/3^rd^ trimester: The experts failed to reach a consensus for 1^st^ line strategies. As 2^nd^ line strategies continuation of the LAI antipsychotic or switching to an oral form (FGA or SGA at the minimum effective dose) is recommended.

#### Elderly patients

**Figure 5:**
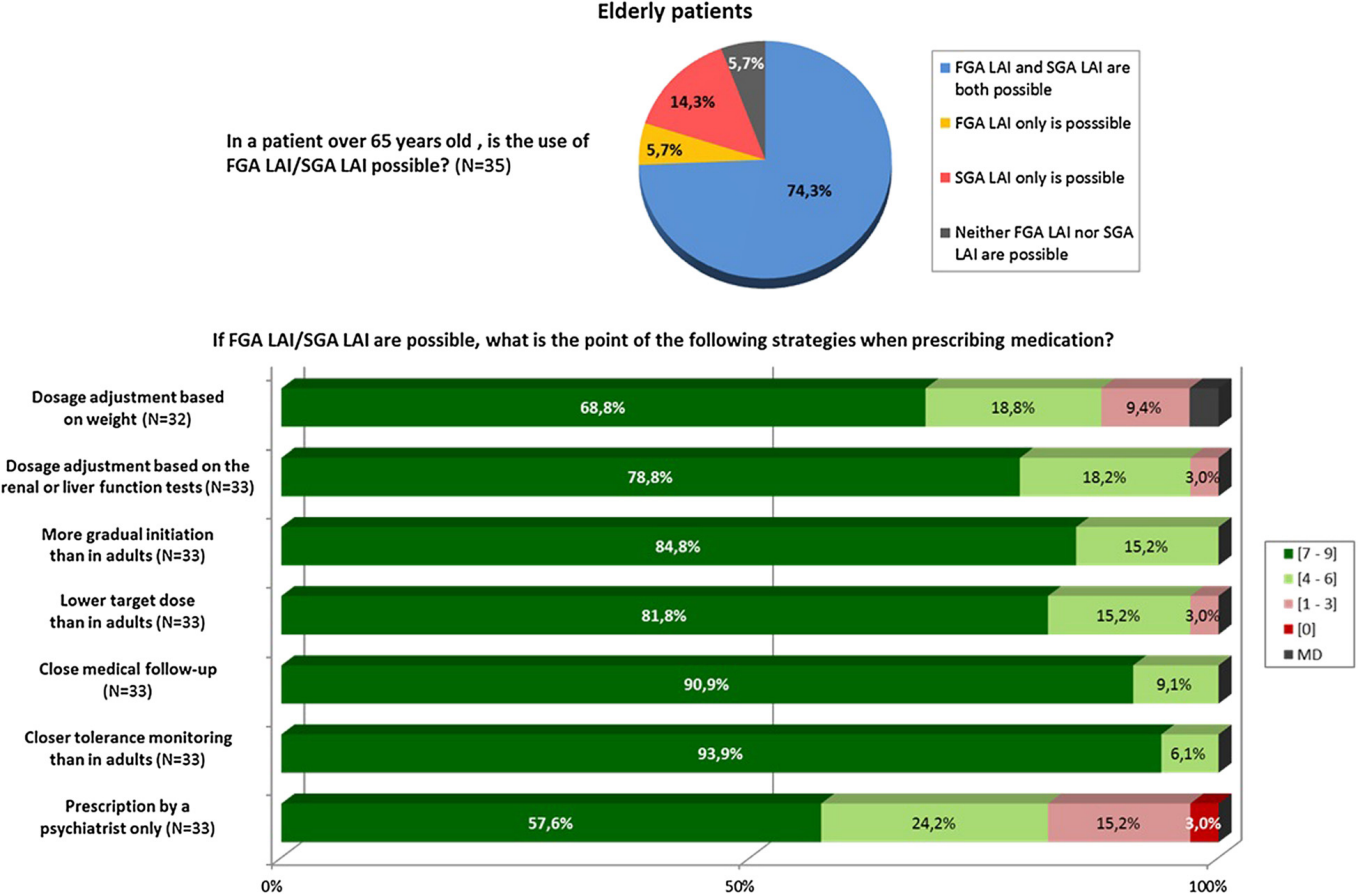
Graphic results of the question about therapeutic strategies in elderly patients.

The relevant question from the survey with the experts’ answers are given in Figure 5.

In elderly patients over 65 years, the use of an LAI antipsychotic is possible. Certain precautions are recommended as 1^st^ line strategies when prescribing an LAI treatment:

Dosage adjustment according to weight, liver or renal function tests.

A longer titration than in adults with a lower “target” dose.

Close medical follow-up (strategy of choice).

Closer tolerance monitoring than in adults (strategy of choice).

Prescription only by a psychiatrist.

#### Subjects in precarious situations

In subjects in a precarious situation, the use of an LAI SGA is recommended as 1^st^ line treatment (LAI FGA as 2^nd^ line treatment).

#### Subjects incarcerated in prison

With incarcerated patients, the use of an LAI antipsychotic can be considered. This prescription does not differ according to the length or the place of incarceration.

The psychiatric indications are the same as for the non-incarcerated population, with the difference being that LAI SGA appears as the treatment of choice for schizophrenic and delusional disorders.

The presence of the following clinical characteristics (aggressiveness, previous history of risk for others) guides the therapeutic choice in favour of an LAI FGA or an LAI SGA in schizophrenic disorders or towards an LAI SGA in bipolar disorders (1^st^ line strategies).

## Discussion

The main interest of our work is to help clinicians make the choice of using an LAI antipsychotic in specific clinical circumstances, using the methodology of consensus-based guidelines (CBG).

### Evidence-based guidelines vs. consensus-based guidelines

Most guidelines for the treatment of psychiatric disorders are evidence-based guidelines (EBG) [11,20]. However, recommendations cannot be established if there is no evidence available, in which case, CBG methodology can be used. The French National Health agency [19] recommends the Formal Consensus method when two of the following conditions are met:

No or insufficient level of evidence addressing the question.

Possibility to decline the topic in easily identifiable clinical situations.

Need to identify and select the strategies deemed appropriate by an independent panel from amongst several alternative options.

This method is very close to the Expert Consensus Guidelines methodology and has been applied to a variety of psychiatric disorders [4,21-27].

Combining EBG and CBG methodologies may help clinicians to have a real evidence-based clinical practice, including both clinical expertise and scientific evidence [20].

In the field of LAI antipsychotic use and management, CBG methodology appears to be particularly appropriate. Evidence concerning LAI antipsychotic efficacy and tolerability exists but it is lacking in many areas (i.e. indications or preferential patient profiles, a ranking system between LAI antipsychotics, the introduction stage, process for switching, medication management, specific populations…). CBGs allow the clinician to be led by recommendations that bear a closer relation to the characteristics of the patients followed in clinical practice than to the restrictive inclusion criteria of randomized-controlled trials [20].

### Indications of LAI antipsychotics

According to our experts’ panel, LAI antipsychotics are recommended as first-line treatment in various psychiatric disorders:

Schizophrenia.

Schizoaffective disorder.

Delusional disorder.

But also as second-line treatment in:

Bipolar disorder.

Personality disorder.

If their use in schizophrenia is common and supported by evidence [5-7,28], their use in bipolar disorder is less obvious. Nevertheless, several placebo-controlled relapse prevention studies have shown the efficacy of risperidone microsphere as a monotherapy or as an adjunctive therapy to lithium or valproate in bipolar I patients [29]. In September 2011, and based on this data, the Food and Drug Administration Agency approved risperidone microsphere as a long-term treatment for bipolar I disorder. Scientific literature is currently limited to risperidone microsphere but the development of new drugs should allow further studies with LAI SGA as maintenance treatment for bipolar disorder.

The use of LAI antipsychotics in other indications (schizoaffective disorder, delusional disorder, personality disorder) is not based on evidence for these populations but is instead based on the clinical experience of our experts’ panel. If scientific evidence is required then the sharing of this experience can be considered as a real support for the clinical use of these compounds.

### Use of LAI antipsychotics during the different phases of the illness

In recent years the interest of using LAI SGA in the early phase of schizophrenia has increased because the duration of untreated psychosis is associated with the prognosis of the illness [30]. Recent studies have underlined the fact that their use, as early as the first psychotic episode, offers many advantages in terms of efficacy, tolerance and improved adherence [31-33]. The available literature presents a weak level of evidence (open label, post-hoc analysis, and small sample) and placebo-controlled studies are needed. The formalized consensus of our experts’ panel is consistent with these preliminary results and recommends LAI SGA after the first schizophrenic episode.

The extension of this data to the first manic episode in bipolar disorder could be assumed but, to date, no data has emerged that compares the effect of the early introduction of oral or LAI antipsychotics on the course of the illness. This is probably the reason why the experts’ panel did not recommend LAI SGA in the early course of bipolar disorder as a maintenance treatment.

### What is the specific clinical profile of patients using LAI antipsychotics in clinical practice?

Our experts’ panel considers that LAI antipsychotics should be used with any patients with schizophrenia for whom maintenance antipsychotic treatment is indicated. This is consistent with the results of a survey conducted among psychiatrists from Europe, Middle-East and Africa, in which clinicians considered switching to or adding an LAI antipsychotic as the preferential pharmacological approach for addressing adherence problems [34].

LAI antipsychotics have long been viewed as a treatment that could only be used for a small subgroup of patients with non-compliance, frequent relapses or who pose a risk to others. A cluster analysis of French and German studies, surveying psychiatrists about patient attributes that potentially influence their qualification for depot treatment, identified two clusters of patients [35,36]. Cluster I corresponded to the classical patient profile in whom depot forms are used (past history of relapse and poor compliance with oral forms). Cluster II was more unexpected and included patients with high levels of insight and of therapeutic alliance. The usefulness of depot formulations compared with oral treatment in terms of relapse prevention is not demonstrated in this population [35,36]. However, even limited gaps of treatment with oral formulation (11–30 days a year) is enough to increase the risk of relapse by 2.81 in patients with schizophrenia [37]. The identification of the two clusters, replicated in numerous countries, is consistent with the recommendation of our experts’ panel.

Considering the risks associated with non-compliance in bipolar patients [38,39], the experts’ panel recommends LAI antipsychotics as a second-line treatment in bipolar disorder.

### LAI FGA vs LAI SGA

If the superiority of LAI antipsychotics versus placebo, in terms of relapse prevention, has been demonstrated [5,28] for schizophrenia, no study compares the LAI SGA versus LAI FGA.

We can only extrapolate the results from studies on oral antipsychotics. Some individual oral SGA (amisulpride, clozapine, olanzapine, risperidone) were better in overall efficacy in patients with schizophrenia than oral FGA [17]. Other oral SGA were no more effective, even for negative symptoms. However, a meta-analysis which considered all oral SGA as a single group demonstrated that they were associated with fewer relapses, less treatment failures and fewer hospitalizations in the long-term treatment of schizophrenia [40]. Oral SGA induced fewer extrapyramidal side effects than oral FGA but some SGA induced more weight gain or metabolic side effects than oral FGA [41,42]. Tolerance profiles of oral SGA are more mixed and require the characteristics of each molecule to be taken into account on an individual basis.

In a one-year observational study including 1859 patients diagnosed with schizophrenia, an adjusted Poisson regression analysis showed that the use of risperidone microsphere was associated with a lower rate of hospitalization compared to the use of other LAI FGA [43].

So, with no evidence available, the experts’ panel recommended that the clinician’s decision-making process takes into account the benefit/risk balance and prioritizes LAI SGA (except for olanzapine pamoate, due probably to the risk of post-injection syndrome [44]) over LAI FGA, according to patient tolerance.

### Use of LAI antipsychotics in clinical practice guidelines

The management of LAI antipsychotics in clinical practice can sometimes be complex for clinicians and there are limited data or recommendations in the literature. Our guidelines try to propose practical recommendations to facilitate the introduction, switching and management of LAI antipsychotics in the different phases of schizophrenia or bipolar disorder.

Indeed, the current EBG for biological treatment of schizophrenia and bipolar disorder [8-10,45-53] propose few recommendations concerning LAI antipsychotics.

Most of them recommend the use of LAI antipsychotics only for patients with non-adherence, frequent recurrence or who prefer this formulation. The conditions of use and management are not, or are only briefly, described. LAI antipsychotics are presented separately from the oral medication strategies (except for the CANMAT guidelines in bipolar disorder).

The main reasons given in explanation for the limited number of recommendations regarding LAI antipsychotics are related to the lack of long-term studies and the lack of high-quality evidence comparing LAI SGA to oral SGA. Perhaps the follow-up period, lasting a year or less, may have been too short to reveal the longer-term benefits of depot treatment versus oral form [9,46].

However, in our opinion, the current criteria for level of evidence are probably not adapted to the studies dealing with LAI antipsychotics. Indeed, randomized-controlled trials have a major selection bias and cannot assess the potential adherence benefits of LAI formulations (non-compliant patients do not participate in a trial and those who accept to be included are the most compliant). Therefore, it can be difficult to demonstrate the benefit of LAI antipsychotics compared with oral antipsychotics. Future studies with LAI antipsychotics should combine the strengths of the different study designs (randomized-controlled studies, mirror-image studies or cohort studies).

In addition to these EBG, there are some CBG focusing on the use and management of LAI formulations for the treatment of schizophrenia [4,27,54-57].

The first guidelines, published in 1998, already recommended that LAI FGA should be considered for “*any patients with schizophrenia for whom long*-*term treatment is indicated*” [54]. However, with the emergence in the years that followed of oral SGA, which are better tolerated compared to FGA, most of the guidelines have been in favour of the use of the oral formulation. Since the market authorization (2002) of the first LAI SGA (risperidone microsphere), two other specific guidelines concerning LAI antipsychotics [27,57] have been proposed. These guidelines recommended LAI SGA as first-line treatment for patients who request the long-acting formulations. Their use after the first schizophrenic episode or for patients who are stable with oral antipsychotics has been discussed.

In 2009, Velligan et al. published expert consensus guidelines about adherence problems in patients with serious mental illness [4]. Use of LAI antipsychotics was a personal choice for patients with frequent relapses associated with non-adherence, relapses because they stopped taking the medication, or because they expressed a preference for the LAI formulation.

The *Association des médecins psychiatres du Québec* (AMPQ) has also recently developed guidelines concerning LAI antipsychotics with a decisional algorithm, which places the depot formulation in every step of treatment as soon as possible [56].

## Conclusion

The evolution of the therapeutic arsenal for schizophrenia and bipolar disorder, with the development of LAI FGA, then oral SGA, and finally LAI SGA, probably explain the difficulties in changing the prescribing practice for clinicians. Some clinicians consider LAI antipsychotics to be coercive, stigmatizing, unacceptable for patients or impossible to stop immediately when side effects occur [4,13,15]. The negative attitudes of psychiatrists toward LAI antipsychotics mean they require a high level of evidence that depot formulation is clearly superior as a maintenance treatment to oral antipsychotics [58]. Negative beliefs towards depot formulations could be decreased using, as is recommended, shared decision-making and minimizing the experience of patient coercion. The current and future availability of a larger number of LAI SGA (aripiprazole, paliperidone, olanzapine, risperidone) should allow the clinicians to embrace depot treatments more easily.

If the interest in LAI treatments has been shown in terms of a decreased risk of relapse in patients with schizophrenia, studies are still required that are adapted, from a methodological point of view, to the assessment of LAI antipsychotics, particularly after the first psychotic episode.

In an evidence-based clinical approach, psychiatrists should be systematically offering to all patients that require long-term antipsychotic treatment, through shared decision-making, an LAI antipsychotic as a first-line treatment (key points are summarized in Appendix 3).

## Appendix 1: Scientific support of the project

### Initiation of the formal consensus guidelines

•French Association of Biological Psychiatry and Neuropsychopharmacology (*Association Française de Psychiatrie Biologique et Neuropsychopharmacologie* - *AFPBN* -).

### Coordination

•Professor Pierre-Michel Llorca/Doctor Ludovic Samalin.

### Project scientific committee

•Doctor Mocrane Abbar.

•Professor Philippe Courtet.

•Professor Pierre-Michel Llorca.

•Doctor Sebastien Guillaume.

•Doctor Ludovic Samalin.

•Sylvie Lancrenon.

### Independent scientific committee (ISC)

•Professor Emmanuel Haffen.

•Professor Christophe Lançon.

•Professor Pierre Thomas.

## Appendix 2: list of experts

ALAMOME Isabelle, ATTAL Jérôme, BARTOLI Jean-Luc, BEAUFILS Béatrice, BELZEAUX Raoul, BILLARD Stéphane, BOTTAI Thierry, CANCEIL Olivier, CAPDEVIELLE Delphine, CHARLES Eric, CHEREAU-BOUDET Isabelle, COUSIN François-Régis, De BEAUREPAIRE Renaud, DELAMILLIEURE Pascal, DELAUNAY Vincent, DUFUMIER Emmanuel, FREMONT Patrick, GIORDANA Bruno, GIORDANA Jean-Yves, GIRAUD-BARO Elizabeth, GUILLAUME Agnès, HODE Yann, LACAMBRE Mathieu, LOMBERTIE Emile-Roger, MARON Michel, MEARY Alexandre, MISDRAHI David, MONIE Jacques, MURRY Pierre, NOURRY Patrick, NUBUKPO Philippe, PAULET Catherine, PETIT Marion, PICARD Valérie, PRETERRE Philippe, PROSPERI Antoine, SAUTEREAU Marie, TALEB Mohammed, TRYSTRAM-VACHERON Marie-Noëlle, VIALA Annie, VILAIN Jeanne, ZIMMERMANN Marie-Agathe.

## Appendix 3: key points

1. Long-acting injectable (LAI) antipsychotics are indicated in patients with schizophrenia, schizoaffective disorder, delusional disorder and bipolar disorder.

2. LAI second-generation antipsychotics (SGA) are recommended as maintenance treatment after the first episode of schizophrenia. LAI first-generation antipsychotics (FGA) (depot neuroleptics) are not recommended in the early course of schizophrenia and must be avoided in bipolar disorder.

3. LAI antipsychotics have long been viewed as a treatment that could only be used for a small subgroup of patients with non-compliance, frequent relapses or who pose a risk to others. The panel considers that LAI antipsychotics should be considered and systematically proposed to any patients for whom maintenance antipsychotic treatment is indicated.

4. According to their efficacy and tolerability:

* LAI SGA are recommended as first line and LAI FGA as second line in the maintenance treatment of schizophrenia.

*LAI SGA are recommended as second line as a monotherapy to prevent manic recurrence or in combination with a mood stabilizer to prevent depressive recurrence in the maintenance treatment of bipolar disorder.

5. In order to improve the acceptance and understanding of the benefits of an LAI antipsychotic, it is recommended to deliver to each patient specific information concerning the advantages and inconveniences of the LAI formulation, in the framework of shared decision-making.

6. Process for switching to LAI antipsychotic. Two main situations are identified:

* Switch from an oral antipsychotic:

* Prescribe the oral formulation of the antipsychotic to establish tolerability/efficacy.

* Use an initial dose of the LAI antipsychotic equivalent to oral form.

* Switch from another LAI antipsychotic:

* Use several test doses of the oral formulation of the LAI antipsychotic if patient has never taken this medication previously (to rule out hypersensitivity).

* Introduce the new LAI antipsychotic at the scheduled period of the next injection.

* Use an initial dose of the LAI antipsychotic equivalent to the previous LAI.

7. Medication management:

* Reminders of injection date must be used to improve compliance.

* First line: phone call and diary.

* Second line: letter or text message.

* Coordinate the dates of medical consultations with the scheduled dates of LAI antipsychotic injections.

* Respect the prevention of local complications when administrating LAI:

* Administered by competent/training professional (nurse, psychiatrist, GP),.

* Check the length of needle and penetrate the deep muscle tissue,.

* Select the injection site according to patient preference,.

* Propose systematically a local anaesthetic to reduce pain at the injection site.

* The change of the injection site, for each injection, can be evoked.

## Abbreviations

FGA: First-generation antipsychotic; SGA: Second-generation antipsychotic; LAI: Long-acting injectable; ECT: Electroconvulsive therapy; CBG: Consensus-based guidelines; EBG: Evidence-based guidelines.

## Competing interests

Pr Llorca, Pr Courtet and Dr Abbar have received grants and served as consultant or speaker for the following entities: AstraZeneca, Bristol-Myers Squibb, El Lilly, Janssen-Cilag, Lundbeck, Otsuka, Sanofi-Aventis and Servier. Dr Samalin has received grants and served as speaker for the following entities: AstraZeneca, Bristol-Myers Squibb, El Lilly, Lundbeck, Otsuka and Sanofi-Aventis. Dr Guillaume has no conflict of interest.

## Authors’ contributions

PML and LS have been involved in drafting the manuscript. SL has made substantial contributions to the acquisition and analysis of data. All authors have made substantial contributions to the conception, design and interpretation of data, have been involved in revising the manuscript critically for important intellectual content and have given final approval of the version to be published.

## Pre-publication history

The pre-publication history for this paper can be accessed here:

http://www.biomedcentral.com/1471-244X/13/340/prepub
